# Therapeutic Garden With Contemplative Features Induces Desirable Changes in Mood and Brain Activity in Depressed Adults

**DOI:** 10.3389/fpsyt.2022.757056

**Published:** 2022-04-07

**Authors:** Agnieszka Olszewska-Guizzo, Anna Fogel, Nicolas Escoffier, Angelia Sia, Kenta Nakazawa, Akihiro Kumagai, Ippeita Dan, Roger Ho

**Affiliations:** ^1^Institute for Health Innovation & Technology (iHealthtech), National University of Singapore, Singapore, Singapore; ^2^NeuroLandscape Foundation, Warsaw, Poland; ^3^Singapore Institute for Clinical Sciences, Agency for Science, Technology and Research (A^*^STAR), Singapore, Singapore; ^4^National Parks Board, Centre for Urban Greenery and Ecology, Singapore, Singapore; ^5^Department of Psychological Medicine, Yong Loo Lin School of Medicine, National University of Singapore, Singapore, Singapore; ^6^Applied Cognitive Neuroscience Laboratory, Faculty of Science and Engineering, Chuo University, Tokyo, Japan

**Keywords:** depression, nature, brain activity, therapy, urban green space, therapeutic garden, major depressive disorder, contemplative landscape

## Abstract

The therapeutic values of contact with nature have been increasingly recognized. A growing body of evidence suggests that a unique subcategory of “contemplative landscapes” is particularly therapeutic. Previous studies predominantly focused on observational designs in non-clinical populations. It is not known if these effects can be extrapolated to populations suffering from depression, and experimental designs need to be utilized to establish causality. We examined the effects of *in-situ* passive exposure to three urban spaces on brain activity, namely a Therapeutic Garden with high Contemplative Landscape scores (TG), Residential Green (RG) and Busy Downtown (BD), and self-reported momentary mood in adults aged 21–74 (*n* = 92), including 24 clinically depressed and 68 healthy participants. Portable, multimodal electroencephalography (EEG) and functional near-infrared spectroscopy (fNIRS) systems were used to record brain activity, and a Profile of Mood States (POMS) questionnaire was used to record mood before and after exposure. We tested the interactions between the site, time and group for the mood, and between site and group for the neuroelectric oscillations and brain hemodynamics. Self-reported pre- post-mood was significant only at the TG (*p* = 0.032) in both groups. The lowest Total Mood Disturbance (TMD) was reported at TG and the highest in BD (*p* = 0.026). Results from fNIRS indicated marginally significant lower oxy-Hb in the frontal region at TG as compared to BD (*p* = 0.054) across both groups. The marginally significant effect of site and group was also observed (*p* = 0.062), with the Clinical group showing much lower oxy-Hb at TG than Healthy. The opposite pattern was observed at BD. EEG results showed differences between Healthy and Clinical groups in the Frontal Alpha Asymmetry (FAA) pattern across the sites (*p* = 0.04), with more frontal alpha right in the Clinical sample and more left lateralization in the Healthy sample at TG. Temporal Beta Asymmetry (TBA) analyses suggested that patients displayed lower bottom-up attention than Healthy participants across all sites (*p* = 0.039). The results suggest that both healthy and depressed adults benefitted from exposure to TG, with possibly different pathways of mood improvement. Visiting therapeutic nature with contemplative features may provide valuable support for the treatment of depression in clinical populations and a self-care intervention in non-clinical populations.

## Introduction

There is a growing body of evidence supporting the broad spectrum of mental health benefits associated with exposure to specific natural environments (1, 2). In environmental psychology and landscape architecture research, these environments have been called salutogenic (3), contemplative (4), tranquil (5), restorative (6), sensory (7), and therapeutic (8), among other names. Despite this semiotic abundance, they are all designed to induce a positive health response through soothing contact with nature and are thus considered “therapeutic.” Landscape architecture research, however, pointed out specific physical attributes which may amplify the therapeutic effect of these spaces. These attributes have been grouped into seven key categories (Landscape Layers, Landform, Vegetation, Color and Light, Compatibility, Archetypal Elements, and Character of Peace and Silence) and incorporated into an expert-based tool for the visual assessment of urban green space, called a Contemplative Landscape Model (CLM) (9). Research findings showed that visiting therapeutic gardens (for simplicity, this term will be used to refer to therapeutic green spaces) can improve mood (10), regulate emotions (11), reduce stress (12), reduce body inflammation (13), and improve quality of life (14). Research also shows that the therapeutic garden scenes with high CLM scores could be superior in delivering health benefits than standard green space (15). This further suggests that exposure to therapeutic gardens with certain contemplative features can support the treatment of depressive disorders, the most common mental illness worldwide. Between 1990 and 2017, incidents of depression worldwide increased by 50% (16). This figure is expected to grow significantly due to the COVID-19 pandemic (17, 18). Additionally, it has been established that city living increases the risk of developing depression (19, 20). Given the limitations of treatment accessibility and a growing number of people suffering from depression, testing new self-care interventions, such as visiting therapeutic gardens, appears to be a feasible and justified approach to support traditional treatment methods.

There have been many observational and epidemiological studies that allude to the therapeutic effects of gardens on mental health, though advanced experimental designs with robust methods of measurement are required to establish causality and unravel mechanistic pathways. Methodological and technological advancements in brain imaging allow for running *in-situ* experiments with a high level of ecological validity, during the actual exposure to sites of interest, as opposed to observational or self-reported outcomes, or more traditional laboratory-based experiments. This is thanks to non-invasive, portable, and reliable devices such as functional near-infrared spectroscopy (fNIRS) and electroencephalography (EEG). They offer a signal quality while recording in the field that is comparable with that of a fully-controlled lab environment (21–23). This is an emerging field of research that offers an opportunity to identify the specific pathways between therapeutic effects of nature on mental health and individual differences in brain pattern activity in the general and clinical populations.

In our preliminary study we found a pattern of brain activity suggestive of positive emotions and relaxation during passive exposure to the Therapeutic Garden among 24 healthy adults (15). Our findings stimulated further research on a larger sample, including the comparison of brain activity between healthy and depressed adults. Our aim was to build on the earlier findings and explore the effects of exposure to three different sites (two green spaces, but with different landscape quality, and one control—a busy urban environment with no greenery) on self-reported and direct measures of mood, and to examine whether healthy participants differed in their response from the participants with depression. For direct measures of mood, we used EEG and fNIRS. We expected that the difference in the alpha oscillations between the sites would follow the preliminary study findings. In addition, we added beta power band and fNIRS analyses to explore the attention patterns and hemodynamic response in these spaces, respectively. We further expected to see significant differences in neuro-electrical and hemodynamic signatures of the beneficial effect of passive exposure to therapeutic nature between the Clinical group of depressed individuals and the Healthy group. We hypothesized that passive exposure to therapeutic gardens can significantly improve the mood of all visitors: healthy adults as well as those suffering from the mood disorders, such as depressive disorder. Furthermore, we expected that this effect would not be observed in green spaces where physical characteristics of the landscape design are different.

## Materials and Methods

### Participants

We recruited 92 right-handed adults aged between 21 and 75 years old, 52 of which were females. Healthy participants (*n* = 68, 37 female) were recruited through word of mouth. The Clinical group (*n* = 24, 15 female) consisted of 17 patients diagnosed and treated for depressive disorder, who were recruited at the National University Hospital's Department of Psychological Medicine. Seven participants initially recruited for the Healthy group, who reported previous treatment for depression and clinically concerning moderate or severe depression, were subsequently reclassified to the Clinical group by the expert psychiatrist, even though at the time of recruitment they were not patients of the clinic. All participants were reimbursed for their time.

### Site Selection

To test the hypothesis, three locations with different composition of natural and built elements were selected in the highly urbanized city-state of Singapore: (1) a Therapeutic Garden (TG), (2) a residential green public space designed without special therapeutic considerations (RG), and (3) an urban space with negligible greenery in a Busy Downtown area (BD). At each of the three locations, three distinct points of view were selected to represent different vistas typical to that location. Selected views were blindly coded by four landscape architecture experts according to the Contemplative Landscape Model (CLM), and an average score was computed for each site, in line with the recommended practice (9). Landscapes with higher CLM score have more contemplative landscape characteristics aggregated within the view and are more likely to induce the positive changes in mood and stress reduction. The CLM scores range from one to six points, and previous research has suggested that a score of 4.45 points and above can induce positive changes in brain activity (24).

The first site, TG, is part of a larger park called HortPark. It is the first specialized garden to offer both an activity area for inclusive nature-based group programs and a healing space designed to foster restoration from stress and mental fatigue. This intimate green space was carefully curated with vegetation that comprises wide-canopy trees and groupings of plants to stimulate the senses. Benches are aptly spaced, with views toward softly fascinating landscapes in the surroundings, facilitating nature contemplation and relaxation. Calming features, such as a watermill and chimes, are installed to promote a sense of peace and wellbeing. TG was assigned a high Contemplative Landscape Model [CLM (9)] score−4.63 points on the 1–6 point scale.

The second site, RG, is a green roof area of a public housing residential estate. In Singapore, the majority of the population lives in public housing commonly called HDB (from the Housing and Development Board, which is the name of the government agency in charge of their development). The HDB estates consist of high-rise and high-density blocks with multiple common green spaces and facilities accessible to the public. The experimental site was selected to be at Casa Clementi HDB Estate, on the recreational green roof over the underground parking area, consisting of paths among lush greenery, garden shelters and pergolas, community farm, biking trail, and children's playground. This site received overall 2.7 points on the CLM.

The third site was selected to represent the fully urbanized landscape of a busy downtown area with little to no natural elements. It is located in the central district near Chinatown and the busy Mass Rapid Transit interchange station. The views selected for this site are dominated by crowds of people, streets with high traffic, and lots of infrastructure. This site was not assigned a CLM score, as it is not a green space.

**Figure 1:**
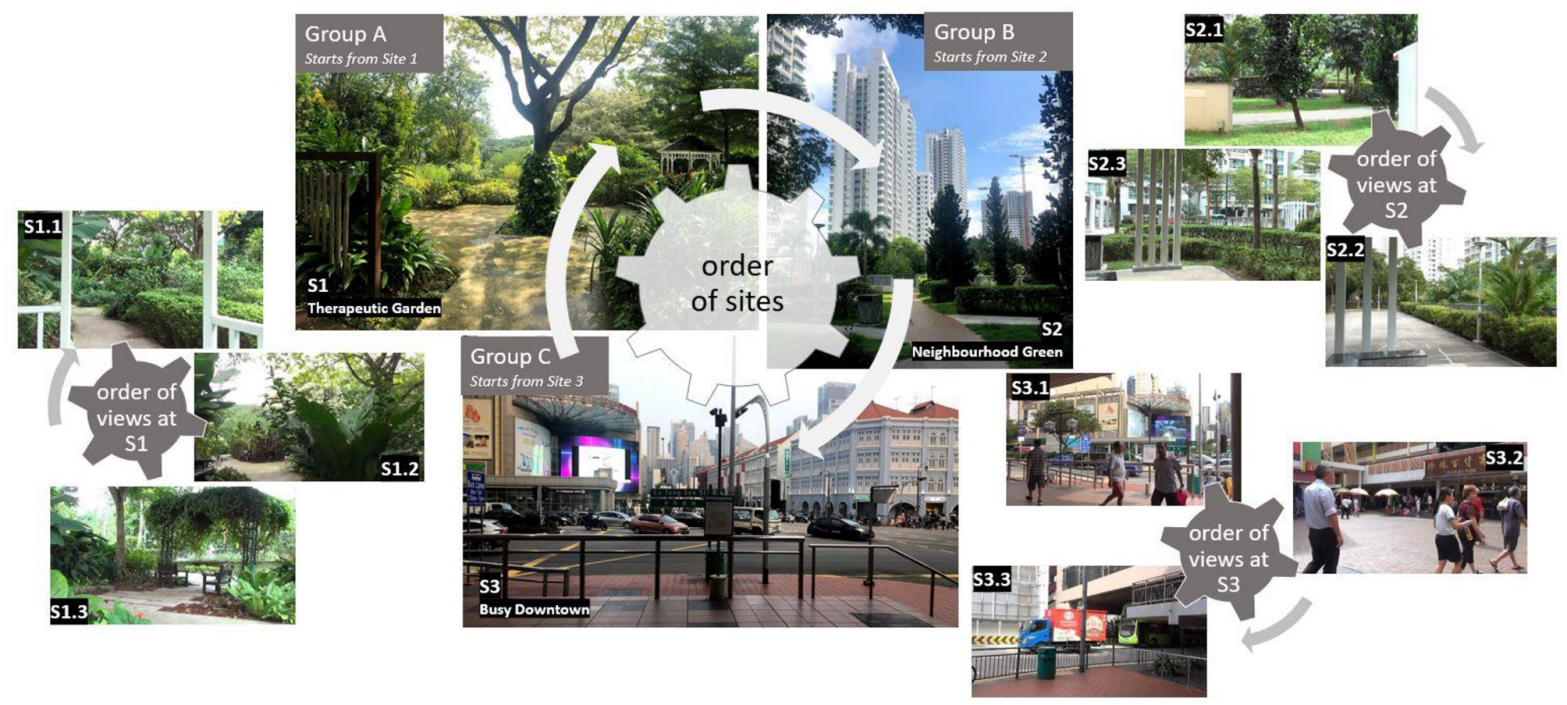
Selected sites and the order of visits.

Scenes, locations, and walking areas were selected in shaded areas to avoid excessive sun exposure. To avoid order bias, we randomized both the order of the location visits and the order of site exposure within each location. Randomization of the site and view order was based on the Latin square design (simple rotation) (25), so that an equal number of participants started the experiment at each site and each view. The randomization scheme is presented in Figure 1.

### Methods

To measure momentary mood changes we used a paper-based questionnaire, Profile of Mood States [POMS (26)], one of the most widely used affective psychometric instruments in environmental psychology (2). It consists of 40 adjectives that measure the Total Mood Disturbance (TMD) as well as subscales of mood states: tension, depression, fatigue, vigor, confusion, anger, and esteem-related affect. For each adjective the participant chooses a score on a 0–4 point scale from “Not at all” to “Extremely,” based on how they feel at the moment. POMS showed high reliability (Cronbach's alpha = 0.798) and good validity scores in the outdoor setting (26).

**Figure 2:**
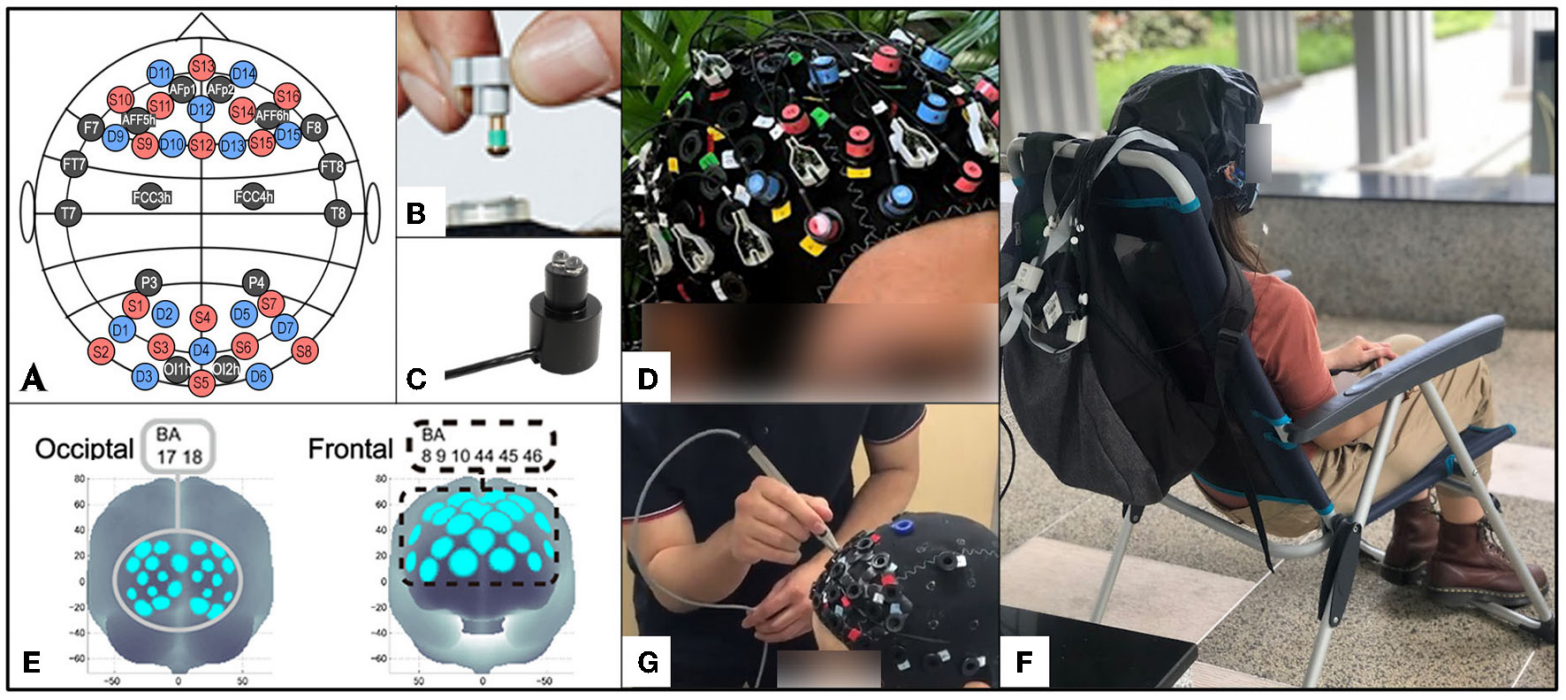
Experimental equipment and setup: **(A)** montage of the multi-modal system with EEG electrodes marked in black, fNIRS sources (S) with red and detectors (D) with blue; **(B)** NIRSport optode with a dual-tip; **(C)** EEG dry active electrode with an adjustable mushroom-head; **(D)** multi-modal NIRSport/EEG V-amp systems mounted onto one stretchable anti-cap, before the light-blocking over-cap placement; **(E)** regions of interest for fNIRS analysis according to Brodmanns Area (BA): occipital (BA: 17, 18) and frontal (BA: 8, 9, 10, 44, 45, 46); **(F)** participant with the brain scanning device mounted on her head, during the passive task; **(G)** digitization of the probe positions using a 3D-digitizer (Fastscan, Polhemus).

To measure the brain neuro-electrical activity and hemodynamics, we used a multimodal setup consisting of EEG, 16-channel V-amp amplifier (Brain Products GmbH, Munich, Germany) with dry active electrodes in modified 10/20 system (Figures 2A,B), and two portable NIRS SPORT devices by NIRx^®^ (NIRx Medical Technologies, LLC, Berlin, Germany), with eight sources and eight detectors (Figures 2A,C) each, combined in tandem mode onto the same stretchable aniCAP supplied by NIRx^®^ (Figure 2D), together with EEG, according to a pre-set prefrontal-occipital montage (Figures 2A,E).

fNIRS is a relatively new brain-imaging technique providing a non-invasive and robust measurement of the light intensity changes (wavelengths between 650 and 1,000 nm) caused by the concentration of oxygenated hemoglobin (oxy-Hb) and deoxygenated hemoglobin (deoxy-Hb) in the brain vessels (27). The brain's neural activity in certain regions triggers an increase in blood flow and volume in those regions, which is disproportionately higher than the metabolic demand (28). Even passive exposure to landscape scenes requires cognitive resources, which can be reflected on the fNIRS scan. In this study, we are interested in observing the regional hemodynamics of the frontal and occipital regions in different sites and groups. The frontal region is one of the most important neuroanatomical structures and plays an important role in emotional processing (29), among other executive functions. The occipital lobe, on the other hand, is commonly associated with visual processing and spatial orientation (30, 31).

EEG records the electrical activity from the cortical regions of interest captured at the scalp, and divides the signal into several power-bands or brainwaves (32). The two most common brainwaves to signal the level of cortical alertness are alpha and beta waves. Alpha brainwaves are of lower frequency (8–13 Hz) and are most prominent during relaxation. Higher frequency beta waves (14–30 Hz) mark attentional processing and increase during task engagement. The first EEG marker examined in this study is Frontal Alpha Asymmetry (FAA). This marker is thought to capture attitudinal and behavioral tendencies toward a set of stimuli, so called approach-withdrawal mechanism (33, 34). A relative increase in alpha power in the right hemisphere (right-sided FAA) has been associated with a positive approach behavioral tendency and more generally reduced inhibition of behavioral and emotional responses (35). Right-hemispheric FAA is observed when the right frontal cortex has greater alpha power than the left frontal cortex, which corresponds to reduced activity in the right and larger cortical resource allocation in the left frontal cortex (36). Conversely, left-hemispheric FAA is characterized by greater alpha power and reduced activity in the left frontal cortex as compared to the right. Notably, previous studies have consistently reported left-hemispheric FAA in patients diagnosed with depression (37–41). However, according to a recent meta-analysis, it is not suitable as a diagnostic measure for major depressive disorder (35). Nonetheless, some work suggests that FAA is relevant to depression treatment. A comparison of depressed participants before and after a 12-week antidepressant treatment highlighted right-sided FAA as a significant predictor of positive treatment outcome, while participants who did not respond to medication showed greater left-sided FAA analysis than participants who did (42). Furthermore, FAA has been shown to be a useful target for neurofeedback used as treatment for depression (43, 44).

The second EEG marker identified as relevant to this study is Temporal Beta Asymmetry (TBA). Right-hemispheric TBA corresponds to more beta power in the right temporal lobe. The temporal region of the right hemisphere is, among other functions, responsible for visual attention (45), interpreting visual information and memory of pictures, visual scenes, and familiar faces (46). Previous studies associated TBA with bottom-up, stimuli driven attention directed at the salient stimuli (47). This bottom-up type of attention is triggered by external stimuli and is opposite to goal-oriented attention. The latter is typical to task-related processing, which, when performed for too long, leads to mental fatigue (48). Bottom-up attention is the central concept of the Attention Restoration Theory (ART), according to which contact with the natural environment leads to the restoration of depleted attentional capacities and to recovery from mental fatigue. In previous studies TBA brain pattern was conceptually linked to “fascination” (24), a key component of a restorative environment according to ART (49, 50).

### Experimental Protocol

The experiment was conducted in the tropical city-state of Singapore, with neither distinct seasonal nor vegetation changes throughout the year. Data was collected between March 2019 and September 2020, during the morning or late afternoon hours of the working week. Participants were blinded to the hypothesis. Experimental sessions were scheduled individually to view at least three scenes within one site. On average, there were 9.1 days between the scheduled sessions (SD = 7.6). After arriving to the scheduled location, participants completed the informed consent and socio-demographic questionnaire, and then POMS before the exposure to each site. Afterwards, they were seated on a chair facing the pre-selected scene and the multimodal EEG-fNIRS brain-scanning apparatus was installed on their head (Figure 2D). Participants were then instructed to put on the white glasses to block the view and then to relax, while the equipment was calibrated and the raw signal recording was initiated. For the EEG recording, the electrode impedance was kept under 100 kΩ throughout the experiment, which is considered an acceptable value for dry electrode systems (51). Signal was recorded at 500 Hz. Cortical hemodynamics were measured with two wavelengths of near-infrared light (760 and 850 nm) and the sampling rate was set at 3.47 Hz.

After 1 min recording of the resting state with the white, view-blocking glasses on, the participants were asked to remove the glasses and passively watch the landscape in front of them for another 1 min. Once this was completed, the 1 min resting state with glasses on and 1 min scene watching was repeated a second time for the same scene. This process was repeated for all three scenes in each location. After the recording for all three scenes was over, the participant completed the post-measurement POMS questionnaire.

Each session lasted from 30 to 45 min depending on the speed of participant's relocation between scenes and smoothness of the machine calibration. Participants were allowed to consume water between individual scans, but not food. Environmental variables [temperature (°C), humidity (%Rh), brightness (Lux), and noise (dBA)] were recorded with a 4-in-1 Environment Meter (CEM, DT-8820) at each scene for each participant to control for confounding variables. The experimental setup is illustrated in Figure 2F.

### Data Processing

POMS scores were calculated by summing the numerical ratings for items contributing to the subscale. The TMD score was calculated by summing up the totals for the negative subscales and then subtracting the totals for the positive subscale, and the constant of 100 was added to the result in order to eliminate the negative values {TMD = [(Ten+Ang+Fat+Dep+Con)–(Era+Fat)]+100}. Air pollution data, more specifically the 24 h Pollutant Standards Index (24 h-PSI) scores, were derived retrospectively from the online resources available at the National Environment Agency website (haze.gov.sg). The brain recordings were processed according to specific steps presented below.

#### EEG Data Processing

The EEG raw data was processed offline in Brain Analyzer 2 software (Brain Products GmbH, Munich, Germany). The signal was filtered with a 50 Hz notch filter, a low-pass at 40 Hz and a high-pass at 0.5 Hz (all were zero phase shift Butterworth filters, order 2). Channels were referenced to an average reference of 16 electrodes and visually inspected for noisy or missing channels. Topographic interpolation of noisy or lost channels was performed where necessary. Ocular artifacts (eye blinks and eye movement) were captured by Independent Component Analysis (ICA) and removed from the data. The signal was epoch time-locked to the onset of the viewing period (1–60 s), and resting baseline data split into matching 59 s long epochs. Each epoch was then split into 1 s equal segments, and noisy segments were removed using an artifact rejection tool. All data underwent Fast Fourier Transformation and were output as power density (μV^2^/Hz). Power density values were then averaged over each condition and alpha (8–13 Hz) and beta (14–30 Hz) bands were extracted. Before further processing all data was baseline-corrected (viewing period–resting baseline) (52, 53).

To compute the FAA values the alpha power on the left frontal lobe (LF, sum of AFp1, AFF5h, F7) and on the right frontal lobe (RF, sum of AFp2, AFF6h, F8) was inserted into the standard equation: (RF–LF)/(RF+LF) (54, 55). Positive FAA values are indicative of greater alpha power on the right frontal lobe as compared to left and negative of greater alpha power on the left frontal lobe compared to the right.

To compute the TBA values, beta power on the left temporal lobe (LT, sum of FT7 and T7) and on the right temporal lobe (RT, sum of FT8 and T8) was inserted into the equation (RT-LT)/(RT+LT). Positive TBA values are indicative of greater beta power on the right temporal lobe as compared to the left. Conversely, the negative TBA values indicate more beta power on the left temporal lobe than on the right.

#### fNIRS Data Processing

Signals reflecting the oxygenated hemoglobin (oxy-Hb) and deoxygenated hemoglobin (deoxy-Hb) concentration changes were calculated in units of millimolar-millimeter (mM-mm) using Homer 3 software (56). We analyzed changes in oxy-Hb signal because of its higher sensitivity to changes in cerebral blood flow than that of deoxy-Hb and total-Hb signals (57–59), its higher signal-to-noise ratio (58), and its higher retest reliability (60). We performed all preprocessing procedures with Matlab 2007b.

Positional data of sources and detectors were obtained for two non-participants of fNIRS measurement (′1 male, ′1 female, both age 24) using a 3D digitizer supplied by NIRx^®^ (Figure 2G). For spatial profiling of fNIRS data, we adopted the probabilistic registration method (61–63), to register the data to the Montreal Neurological Institute's (MNI) (Montreal, QC, Canada) standard brain space. The macro-anatomical labeling was based on Rorden and Brett (64). We divided the brain region following Brodmanns Area (BA) into 2 regions: the occipital area (BA: 17, 18) and the frontal area (BA: 8, 9, 10, 44, 45, 46) as shown in Figure 2E.

Oxy-Hb signals of all channels were corrected for motion artifacts using CBSI method (65). Moving average methods were applied to remove short-term physiological noises in the analyzed data (moving average window: 10 s). The baseline of the task block following was determined by the mean of the last 10 s of the baseline block. From the preprocessed time series data, we computed the oxy-Hb values by calculating the inter-trial mean of the oxy-Hb signals for all the target periods in each site (10–60 s).

#### Statistical Analyses

Initial group comparisons were conducted using *t*-tests and χ^2^ to identify the potential covariates for the main analyses. Participant age and highest attained education level were identified as the potential confounders and included as covariates in the main analyses. In addition, participant ethnic background was also included as a covariate. Although ethnicity was equally distributed between the groups, there was a trend for the participants with non-Chinese ethnic background to have slightly higher BDI scores (Δ = 4, *t* = 1.88, *p* = 0.072) so ethnicity was also included as a covariate in the main analyses. We looked at multiple potential environmental covariates including noise, temperature, humidity, brightness, and total mood disturbance (the average of the individual environmental confounders), as well as individual situational confounders (alcohol intake in the last 24 h, sleep quality and duration, medication). As these factors were not linked to the outcomes and were not more prevalent in either group, and to preserve the degrees of freedom and maximize the power in the analyses, they were not included as covariates in the analyses.

Linear mixed models (LMM) were fitted to examine the differences between the Healthy and Clinical samples in the self-reported mood disturbance (TMD), frontal and occipital hemodynamics (oxy-Hb), FAA, and TBA across the three exposure sites. LMM was deemed most appropriate due to large individual variations in the intercepts. All models tested the main effects of group and site, as well as the interaction between group^*^site. Restricted maximum likelihood estimation was used to fit the models, with identity matrix covariance structure (selected based on the AIC criterion). Random intercepts and slopes were fitted to account for the individual differences in the outcome variables. For the TMD analysis, we explored the main differences between the sites, main differences in change over time (before and after viewing), and group differences between the Healthy and Clinical samples. In addition, we looked at the interaction between the site and time to examine whether both samples showed improvement or worsening of TMD from before to after the viewing depending on the site, and a 3-way interaction between time^*^site^*^group to test whether these changes over time across the sites differed between the samples. Due to signal quality issues, data from 78 participants (55 Healthy and 23 Clinical) were included in the fNIRS analysis. All analyses were computed in IBM SPSS v.26.

## Results

### Descriptive Findings

Sample characteristics are described in Table 1. Participants with depression were younger (*t* = 2.76, *p* = 0.007) and had lower education level (χ^2^ = 5.45, *p* = 0.02). Gender distribution (χ^2^ = 0.18, *p* = 0.67) and ethnicity distribution (χ^2^ = 0, *p* = 1.0) did not differ across the groups. As was expected, the Clinical sample had higher BDI scores (*t* = 7.21, *p* < 0.001), were more likely to have a medical record (χ^2^ = 48.7, *p* < 0.001), and more likely to be medicated (χ^2^ = 52.9, *p* < 0.001).

**Table 1:** Recruited sample characteristics within both Clinical and Healthy groups.

	**Variable**	**Clinical** **(*n* = 24)**	**Healthy** **(*n* = 68)**	**Overall sample** **(*n* = 92)**
Age	Age range	21–54^*^	21–74	21–74
	Mean (Std. dev.)	31 (9.59)	38.79 (17.01)	36.62 (15.77)
Gender	Male	9 (38%)	28 (41%)	37 (40%)
	Female	15 (62%)	40 (59%)	55 (60%)
Mental health record	No psychiatric history	0 (0%)^***^	68 (100%)	68 (73%)
	Depressive disorder	24 (100%)	0 (0%)	24 (27%)
	Comorbidities (anxiety, dysthymia, personality disorder)	7 (29%)	0 (0%)	7 (7.8%)
Medication	Not medicated	11 (45%)^***^	68 (100%)	79 (86%)
	Medicated^a^	13 (55%)	0 (0%)	13 (14%)
BDI-II score	Minimal (0–13 pt.)	6 (25%)^***^	62 (91%)	68 (74%)
	Mild (14–19 pt.)	3 (12.5%)	6 (9%)	9 (9.5%)
	Moderate (20–28 pt.)	6 (25%)	0 (0%)	6 (7%)
	Severe (29–63 pt.)	9 (37.5%)	0 (0%)	9 (9.5%)
	Total score (Std. dev.)	23.4 (10.93)	6.38 (4.5)	10.8 (10.1)
Vision	Normal	9 (38%)	28 (41%)	37 (42%)
	Corrected to normal	15 (62%)	40 (59%)	55 (60%)
Education	Below tertiary	11 (45%)^*^	16 (24%)	27 (29%)
	Tertiary	13 (55%)	52 (76%)	65 (71%)
Ethnicity	Chinese	17 (70%)	44 (65%)	61 (66%)
	Non-Chinese	7 (30%)	24 (35%)	31 (34%)

a *Anti-depressant medication: Desvenlafaxine 50 mg, Escitalopram 10 mg, Fluoxetine (40 mg × 2, 20 mg × 1), Fluvoxamine 150 mg, Mirtazapine (15 mg × 3, 30 mg × 1), Paroxetine 50 mg, Sertraline 50 mg, Venlafaxine 150 mg*.

* *Denotes significantly different between the two groups at 0.05 level*.

*** *Denotes significantly different between the groups at 0.01 level*.

### Profile of Mood States

#### Total Mood Disturbance

**Figure 3:**
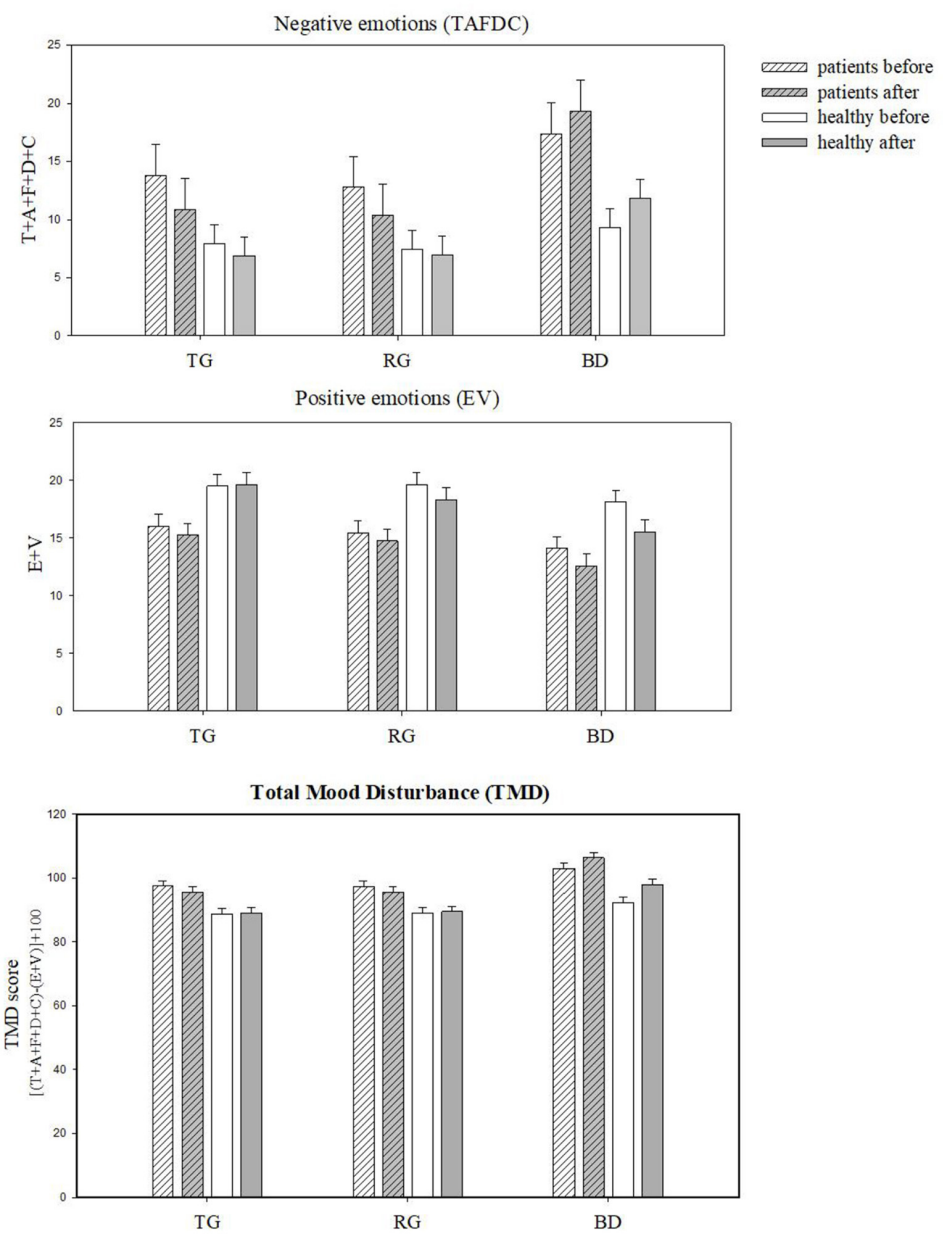
Profile of Mood States scores (±SE) grouped by subscales: negative (TAFDC) and positive (EV) and Total Mood Disturbance (TMD) before and after the exposure to three sites: TG, Therapeutic Garden; RG, Residential Green; BD, Busy Downtown.

There was a significant main effect of site with the highest recorded TMD in the BD (M = 99.1, SE = 1.64), the lowest in the TG site (M = 91.6, SE = 1.64), and in-between recorded in the RG site [M = 92.3, SE = 1.64; *F*_(2, 391.3)_ = 28.7, *p* < 0.001]. The Clinical sample had significantly higher TMD (M = 97.9, SE = 2.64) compared to the Healthy sample [M = 90.8, SE = 1.62; *F*_(1, 75.7)_ = 5.13, *p* = 0.026]. Importantly, the change in TMD from before to after site exposure varied across the sites [*F*_(2, 390.9)_ = 3.47, *p* = 0.32]. Only in the TG the TMD decreased (i.e., mood improved by 2%) after site exposure (Figure 3). In the BD, the TMD increased (mood worsened by ~7%) after site exposure, while in the RG there was no difference from before to after Both the Clinical and the Healthy sample showed similar improvements in TMD after viewing the TG [*F*_(5, 391.0)_ = 0.99, *p* = 0.42].

#### Negative Emotions: Tension, Anger, Fatigue, Depression, and Confusion (TAFDC)

There was a significant main effect of site with the highest negative emotions score in BD [M = 14.472, SE = 1.39; *F*_(2, 415)_ = 20.90, *p* < 0.001]. There was also main effect of group [*F*_(1, 80)_ = 4.351, *p* = 0.04], with significantly higher scores in Clinical (M = 14.09, SE = 2.29) than in the Healthy group (M = 8.41, SE = 1.36). There was also a significant interaction between site and time [*F*_(2, 415)_ = 3.45, *p* = 0.03] suggesting that both groups responded similarly to changes over time across the sites (see Figure 3). Clinical group had the highest negative emotion scores in BD site (M = 18.35, SE = 2.66), significantly higher than the Healthy (M = 21.20, SE = 1.59). Over time the negative emotions decreased significantly in both groups, particularly in the TG (by 22%).

#### Positive Emotions: Esteem-Related Affect and Vigor (EV)

There was a significant effect of time [*F*_(1, 391.83)_ = 10.47, *p* < 0.001] and site [*F*_(2, 392.12)_ = 22.02, *p* < 0.001], suggesting that EV was marginally higher before the viewing (M = 17.29, SE = 0.94) compared to after (M = 16.24, SE = 0.94). EV were the highest in TG (17.82, SE = 0.96) and the lowest in BD (M = 15.17, SE = 0.96). The groups did not differ in EV [*F*_(1, 76.27)_ = 0.69, *p* = 0.41]. No significant interactions were found between group, site and time [*F*_(5, 391.95)_ = 0.507, *p* = 0.771], suggesting that Clinical and Healthy groups did not show changes in positive emotions across the sites as a result of landscape viewing.

### fNIRS Results

**Figure 4:**
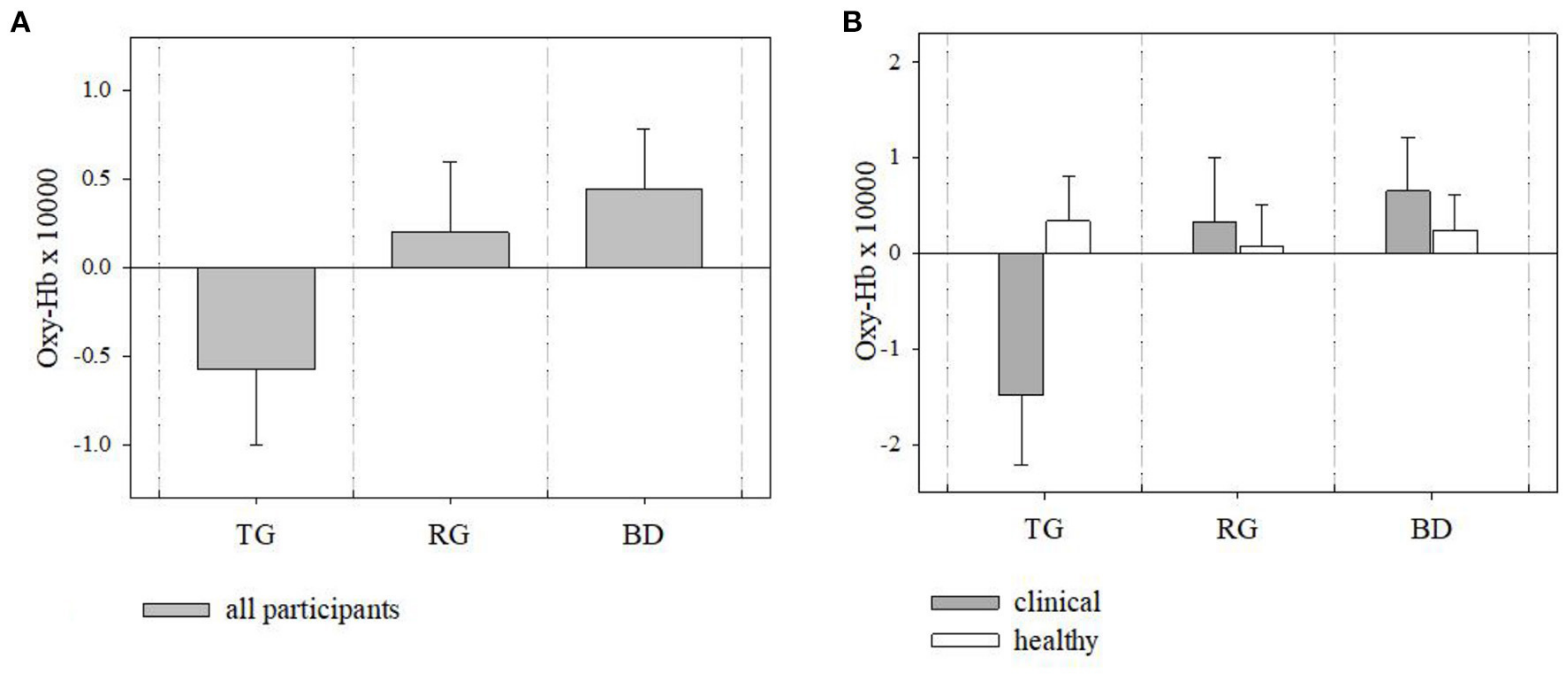
Frontal brain hemodynamics ±SE at different sites (TG, Therapeutic Garden; RG, Residential Green; BD, Busy Downtown); **(A)** differences in levels of oxy-Hb between sites across all participants, **(B)** differences in levels of oxy-Hb between sites and groups. All values multiplied by 10,000 to ease depiction and interpretation.

There was no effect of site observed [*F*_(2, 134)_ = 2.063, *p* = 0.13, Figure 4A]. Pairwise comparisons revealed that oxy-Hb across all participants was lower at TG (M = −0.57, SE = 0.43) as compared to BD (M = 0.45, SE = 0.33), and the difference barely missed statistical significance (*p* = 0.054, Figure 4A). Clinical and Healthy samples did not generally differ in the frontal oxy-Hb [*F*_(1, 63)_ = 0.58, *p* = 0.45]. There was a marginally significant interaction between the group and the site [*F*_(2, 134)_ = 2.84, *p* = 0.06], suggesting that the Clinical and Healthy groups showed a trend toward different patterns of oxy-Hb depending on the site they were at. At TG, the Healthy sample had higher oxy-Hb (M = 0.34, SE = 0.47) as compared to the Clinical sample (M = −1.49, SE = 0.72), and the reverse pattern was observed in the BD, where the Clinical sample had a higher oxy-Hb value (M = 0.65, SE = 0.56) than the Healthy sample (M = 0.24, SE = 0.37, Figure 4B). We also examined oxy-Hb in the Occipital area; however, there were no differences in oxy-Hb across the sites [*F*_(2, 247.98)_ = 0.40, *p* = 0.67], and no differences in the activation between the Healthy and the Clinical samples were observed [*F*_(1, 11.49)_ = 0.82, *p* = 0.38]. There was also no interaction between the group and site [*F*_(2, 247.98)_ = 0.72, 0.49].

### EEG Results

#### Frontal Alpha Asymmetry

**Figure 5:**
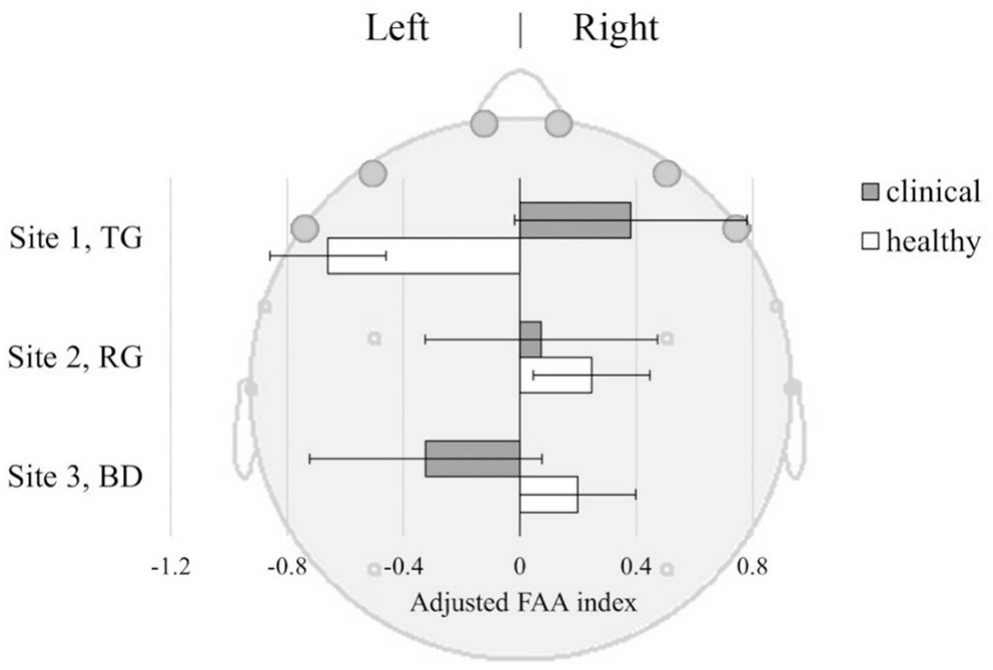
Frontal Alpha Asymmetry (FAA) index ±SE, in Clinical and Healthy samples at three sites: TG, Therapeutic Garden; RG, Residential Green; BD, Busy Downtown. FAA index [(R-L)/(R+L)] was derived from baseline-corrected power density values from the left (AFp1+AFF6h+F7) and right (AFp2+AFF6h+F8) frontal lobes.

The Healthy and Clinical samples did not differ in FAA values overall [*F*_(1, 233)_ = 0.29, *p* = 0.59]. There was also non-significant difference in FAA between the sites [*F*_(2, 233)_ = 0.89, *p* = 0.41]. However, there was a significant interaction between the groups and sites [*F*_(2, 233)_ = 3.27, *p* = 0.040] suggesting that the Healthy and the Clinical samples had different patterns of FAA across the sites. Figure 5 shows mean FAA representing left- and right-sided asymmetry across the sites among the Healthy and the Clinical sample. In the TG the Clinical sample had right-sided FAA while the Healthy sample had left-sided FAA. This pattern was reversed in the BD, where the Clinical sample had left-sided while the Healthy sample had right-sided FAA, though the Confidence Intervals (C.I.) were overlapping. In the RG both samples showed similar patterns of right-sided FAA activation.

#### Temporal Beta Asymmetry

**Figure 6:**
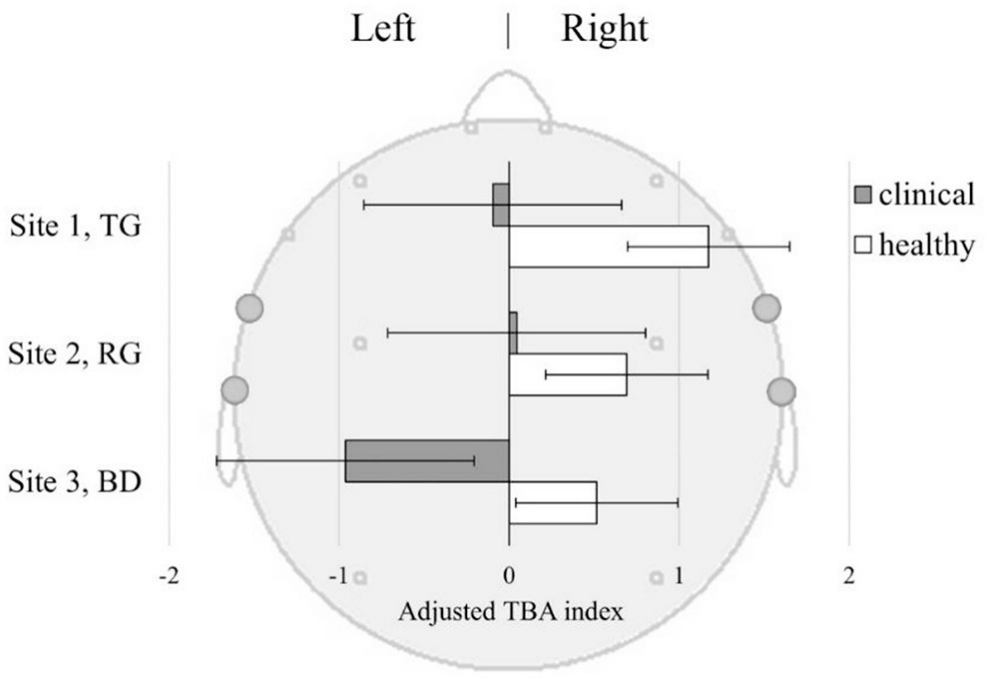
Temporal Beta Asymmetry (TBA) index ±SE in Clinical and Healthy samples at three sites: TG, Therapeutic Garden; RG, Residential Green; BD, Busy Downtown. TBA index [(R-L)/(R+L)] was derived from baseline-corrected power density values from the left (FT7+T7) and right (FT8+T8) temporal lobes.

TBA did not differ between the sites [*F*_(2, 156)_ = 0.44], though the Healthy sample in general had a higher right-sided TBA compared to the Clinical sample [*F*_(1, 75)_ = 4.41, *p* = 0.039]. This was observed irrespective of the viewing site [*F*_(2, 156)_ = 0.24, *p* = 0.79] suggesting that decreased TBA may be inherent to depression (Figure 6). The Healthy sample demonstrated right-sided TBA across all sites, while the Clinical sample only had left-sided TBA in the BD.

## Discussion

The aim of our study was to investigate the impact of a therapeutic garden on mental health, comparing it with exposure to other green and non-green urban spaces. Our second aim was to compare individual differences in the response of healthy and clinical populations suffering from depressive disorder. We expected to observe changes between the groups and between the exposure sites in terms of the brain activity marked with EEG and fNIRS signals as well as between the self-reported mood before and after the exposure. In the study design we used a multimodal brain imaging device measuring two different physiological phenomena EEG for brain electrical reactivity and fNIRS for levels of blood oxidation. Noteworthy, activation patterns must be interpreted with caution and supplemented with other measurements. For example, EEG low alpha activity pattern in one study can be associated with relaxation and creative ideation (66) while in another with boredom and mental fatigue (67, 68). An attempt to conceive meaningful interpretations of the results from two modalities is challenging in a system as complex as the human brain, but bringing us closer to interpret phenomena, specific to only one modality (69).

### Findings From POMS

According to the results from the self-reported mood assessment POMS, exposure to the Therapeutic Garden improved mood (2% drop in the TMD). This effect occurred in both Healthy and Clinical groups, and only at TG and not at the other sites. Residential green did not have any significant effect on mood change, further confirming the hypothesis that not all types of green space exposure have the same therapeutic potential, or in other words, not all green space is the same. What is more, in Busy Downtown the self-reported mood significantly worsened (TMD increased by 7%) from before to after the exposure, suggesting that about 40-minute-long exposure to the busy downtown was a cause of greater mood disturbance in all participants. When looking at the grouped mood states only the negative subscale (TAFDC) showed a site^*^time interaction, with lowest negative emotions at TG and highest at BD site and the largest drop in negative emotions at TG. There were no significant findings in the positive mood subscales (Esteem-related affect and Vigor, EV). Overall, TG exposure significantly improved the general mood of participants in both groups, by reducing the negative affect, indicating its potential universal use as a self-care method.

Previous studies found similar reduction in mood disturbance after a 2-h forest walk in female students (70). Also, after a 30-min urban park exposure the TMD was found to decrease in a study conducted in the UK (71) and in Malaysia (72) among healthy adults. Our findings about TG are then not surprising when considering the healthy populations. However, there was no previous research on depressed people, and according to our findings the Clinical group responded in a similar way as the healthy group. In addition, like in previous studies, we observed increased mood disturbance in both groups in the urban environment, with the Clinical group having a significantly larger jump in negative mood (TAFDC) at that site as compared to a Healthy group. Notably, these negative mood scores were highest in the BD even before the experiment (Figure 3). This suggests that the mood was already disturbed before the experimental protocol has started, perhaps from the moment participants were present at this type of space. At RG, the mood decline or improvement was not observed, even though it is also a green space. Residential areas can be then considered a baseline, the environment from which the mood states can improve or decrease as the individual moves around the urban space along the day. These findings suggest that therapeutic gardens can offset the negative effects of living in the proximity of busy cosmopolitan areas, even in highly populated cities.

### Findings From fNIRS

Considering the outcomes of the brain scanning, we first assessed the regional activity of the frontal and occipital cortex, measured with the levels of oxygenated hemoglobin in the brain vessels. We did not observe a significant effect of site in the frontal lobes (*p* = 0.13), but we observed very close to significant difference in oxy-HB between the TG and BD (*p* = 0.054) across all participants. Similar patterns were described in previous fNIRS studies comparing brain response to urban vs. natural scenes. They found that the natural views triggered lower levels of oxy-Hb concentration and increased oxy-Hb when viewing urban busy scenes in the healthy adult population and associated the lower oxy-Hb with restorative effect (11, 73) and reduced rumination (74). It has been shown that scenes of nature require less cognitive resources and less strenuous attention (75, 76) when compared to busy urban scenes abundant with elements of infrastructure, cars, buildings, and crowds, and our findings seem to support this general premise. When it comes to the differences between the groups, results indicated the marginally significant effect of site^*^group (*p* = 0.06), showing the trend of patterns of brain oxidation in Clinical being different to those observed in the Healthy group. At TG the Clinical sample had much lower frontal oxy-Hb compared to the Healthy sample, and the opposite pattern was observed in the BD. According to the systematic review, oxy-Hb concentration in the frontal cortex of depressed patients are usually significantly lower than in the healthy controls (29). Notably, previous studies with patients with depressive disorder were conducted only in the laboratory setting so it is not known how their brain reacted to different environments. Results of our experiment indicated that patients had more oxy-Hb in the Busy Downtown and lesser in the Therapeutic Garden. fNIRS findings together with the POMS results can then suggest that TG environment induced lowest levels of oxy-Hb in frontal cortex associated with the improved mood, but the effect was stronger for the patients who could also experience reduced rumination. Highest oxy-Hb was observed in patients in the BD, which seem to be associated with increased mood disturbance (Figure 4).

### Findings From EEG

**FAA**. Regarding FAA findings, we did not observe significant difference between the experimental groups and sites considered in isolation. However, the site had a moderating effect on differences in FAA between the healthy and depressed group. The most contrasting difference was observed at the TG. There, we observed right-sided FAA in Clinical group, and left-sided in the Healthy group, suggesting that this environment induces significantly different affective reactivity in Healthy as compared to Clinical group (Figure 5).

Previous research suggests that greater left-hemispheric FAA was found in depressed patients, and we observed that pattern in the Clinical group at the BD location, but not in TG. Previous research also suggests that FAA training and neurofeedback aimed at inducing right-sided FAA in depressed patients has large therapeutic value in depression treatment, as a stimulation of the positive approach-related motivation (43). Furthermore, a comparison of depressed participants before and after a 12-week antidepressant treatment highlighted right-sided FAA as a significant predictor of positive treatment outcome, while participants who did not respond to medication showed greater left-sided FAA analysis than participants who did (42). The right-sided shift in FAA we observed in depressive participants at the TG site suggests that passive exposure to TG induced in our Clinical group a therapeutic pattern aligned with efficacious treatment and a general positive indicator of depression treatment outcome.

Interestingly, the mean FAA of Healthy individuals was left-sided during the TG exposure. While according to the “traditional” interpretation of FAA this could indicate a behavioral inhibition during the scene observation (35), the POMS findings showed that participants experienced mood improvement after spending time at TG. This indicates that FAA and mood measures may capture somewhat distinct mechanisms. This could also be an effect of high variability in FAA data in Healthy participants, unlike in the case of the case with the POMS measures. While mood improved consistently at the TG, behavioral tendencies captured by the FAA were highly variable. A similar dissociation between mood measures and FAA response was observed in the busy downtown environment. Here again, depressed participants showed consistent FAA and POMS indexes, while Healthy participants showed the opposite, a similarly higher variability was observed in their FAA responses. In this environment Healthy participants displayed more approach-related attitude, which suggests that Healthy individuals may be triggered to engage and interact with the social elements in the busy urban space. This may imply people adopt an approach-related attitude toward social interactions in public spaces. In comparison, the peaceful and quiet environment in the TG had lesser trigger for the group to adopt an approach behavior related to social interactions. There are large interindividual differences in the degree to which the environment affects behavioral tendencies in Healthy people, where personal preferences may be at play.

**TBA**. Temporal Beta Asymmetry is an index of how attention is deployed to stimuli. Of special interest here is the fact it can capture attentional patterns linked to the restoration of attention that can be induced by certain types of natural environments. Findings from the TBA analysis show that the Clinical group experienced significantly less of the pattern associated with attention restoration than their Healthy counterparts, regardless of the location (Figure 6). Moreover, in BD the attention restoration as indexed by TBA was significantly lower in the Clinical than in the Healthy group, suggesting that depressed individuals may have experienced extra mental fatigue from prolonged exposure to busy urban environments. Enhanced attention-restoration pattern was observed in the Healthy group at TG; however, the difference between TG and other sites was not significant. This may be related to specificity and duration of exposure as previous studies with self-reported measures of attention restoration, called Restorative Outcome Scale (ROS) (77), found effect on attention restoration after 30 min to 2 h of walking in the park or forest (78, 79), and not after passive exposure (80). Here we used passive exposure of short duration which might have been insufficient to induce the expected patterns.

In summary, the findings of this study provided neuro-psychophysiological evidence of benefits from passive exposure to the Therapeutic Garden for the mental health of individuals with clinically concerning depressive disorders. It further demonstrated that both depressed and non-depressed individuals can benefit from TG exposure, albeit through slightly different pathways. Notably, depressed individuals appeared to benefit consistently from TG exposure. In healthy people, other factors such as presence of social elements might be playing a role in the degree of engagement. Regardless of behavioral tendencies indexed by the frontal asymmetry, the passive exposure to the Therapeutic Garden improved mood in both groups. Moreover, the findings further confirmed that different designs of green spaces (measurable with visual quality assessment tools such as Contemplative Landscape Model) can induce different psychophysiological responses. The study contributed to advancing the knowledge in the field of environmental neuroscience through developing and testing *in-situ* experiment protocols with the use of multimodal EEG and fNIRS brain imaging tools and was an attempt to bridge the findings from the two techniques together.

One limitation of the study is an unbalanced sample. Even though the imbalance was relatively small (27–74%), ideally, there should be equal number of clinical and healthy participants, but the statistics modeling employed mixed effect models which are suited to imbalance datasets (81). Reflective of scientific studies in general, the sample of this study had higher educational attainment than the general population, which limits generalizability of the findings. Also, data collection in the naturalistic setting increases the risk of some unmeasured confounding factors which would be difficult to control for outside of the lab setting. Nonetheless, we controlled for various environmental confounders. fNIRS results must be interpreted with caution as they are highly variable, due to differences caused by the melatonin concentration in the skin (82) and a number of missing data due to low quality of signal. Finally, in this experiment only the momentary changes in mood and brain activity were examined, and only longitudinal data would allow us to determine how long the beneficial effects we observed here can be sustained over time.

## Conclusions

Urban public space of everyday exposure can be seen quite differently by depressed people. Visiting therapeutic nature can constitute an effective and affordable supplement to depression treatment for patients as well as function as a self-care intervention for the healthy population to maintain their mental health. The provision of easily accessible therapeutic gardens to city residents can then be an important strategy for mental health promotion at the city scale and has a potential to offset the negative influence of the busy urban environments on mental health. Future research should focus on replicating the study in various locations and populations, as well as on the assessment of long-term mental health outcomes of exposure to different environments.

## Data Availability Statement

The raw data supporting the conclusions of this article will be made available by the authors, without undue reservation.

## Ethics Statement

The studies involving human participants were reviewed and approved by National University of Singapore Ethics Committee and obtained ethics approvals (NUS-IRB_S-20-12 for 111 healthy and NHG DSRB_2018/01036 for patients). All participants provided their written informed consent to participate in this study.

## Author Contributions

AO-G: conceptualization and investigation. AO-G and NE: methodology. KN and AK: software. AO-G, AF, and NE: validation. AF: formal analysis. RH, AS, and ID: resources. AO-G, KN, and AK: data curation. AO-G and AF: writing—original draft preparation. AO-G, AF, NE, AS, KN, AK, and ID: writing—review and editing. AO-G and KN: visualization. RH and ID: supervision. RH and AS: project administration and funding acquisition. All authors have read and agreed to the published version of the manuscript.

## Funding

This research was supported by the Ministry of National Development Research Fund. This article processing charge was funded by NUS Department of Psychological Medicine (R-177-000-100-001/R-177-000-003-001/R177000702733) and NUS iHeathtech Other Operating Expenses (R-722-000-004-731).

## Conflict of Interest

The authors declare that the research was conducted in the absence of any commercial or financial relationships that could be construed as a potential conflict of interest.

## Publisher's Note

All claims expressed in this article are solely those of the authors and do not necessarily represent those of their affiliated organizations, or those of the publisher, the editors and the reviewers. Any product that may be evaluated in this article, or claim that may be made by its manufacturer, is not guaranteed or endorsed by the publisher.
